# Attitude Toward Using the Triple Combination Bleaching Formula and Related Outcomes: A Cross-Sectional Study

**DOI:** 10.7759/cureus.20542

**Published:** 2021-12-20

**Authors:** Mahdi Al Dhafiri, Mohammed Almutairi, Halal M Alutaibi, Hassan R Aldandan, Fatemah A Albshr, Fatimah S Alkhalifa

**Affiliations:** 1 Department of Medicine, College of Medicine, King Faisal University, Al-Ahsa, SAU; 2 College of Medicine, King Faisal University, Al-Ahsa, SAU

**Keywords:** skin lightening, acne, satisfaction, attitude, kligman’s formula

## Abstract

Introduction

Kligman’s formula is a topical triple combination consisting of hydroquinone, tretinoin, and topical corticosteroid. It has recently become widely popular among the general population for different purposes. Its improper use can lead to unsatisfactory results and unpleasant side effects.

Aim

This study aimed to assess the attitude, satisfaction, and complications related to topical usage of Kligman’s formula among the general population in Saudi Arabia.

Materials and methods

A cross-sectional study was conducted among the general population of Saudi Arabia. A self-administered questionnaire was distributed among the targeted population using an online survey. The questionnaire includes socio-demographic characteristics, assessment of attitude, and satisfaction in using Kligman’s formula. Data were tabulated and cleaned, and all statistical analyses were performed.

Results

A total of 292 participants met the inclusion criteria (26 males vs. 266 females) with a mean age of 26.9 (SD 7.71) years. Nearly 40% of participants showed a positive attitude in using Kligman’s formula, while 46.9% were satisfied with using it. The most common reason for using Kligman’s formula was to lighten the skin (55.8%), while skin redness was the most commonly reported adverse effect. Factors associated with increased attitude and satisfaction were using Kligman’s formula based on a doctor’s prescription and regular follow-up with a dermatologist.

Conclusion

The general population showed an improper attitude toward using Kligman's formula. However, a better attitude and satisfaction rate can be seen among those using Kligman's formula with prescription and those who regularly visit a dermatologist.

## Introduction

Components of the widely known Kligman's formula are 0.1% tretinoin, 0.1% dexamethasone, 5.0% hydroquinone, and hydrophilic ointment. Kligman’s formula is an effective treatment option in treating many dermatological conditions such as melasma and postinflammatory hyperpigmentation [1]. Both postinflammatory hyperpigmentation and melasma are relatively common in Saudi Arabia since there are a high number of people in Saudi Arabia with brown skin (Fitzpatrick skin type III-V) [2,3].

Using skin-lightening products has become highly prevalent among the general population worldwide [4]. In Saudi Arabia, it has shown a prevalence of 56.2% [5]. Among many skin lightening techniques, Kligman's formula is one of the first chosen options, reflecting widespread usage. Other indications of Kligman's formula include dyspigmentation, getting rid of scars, and skin peeling/exfoliation [4,5].

Kligman's formula has been associated with many adverse events (AEs). Most common are erythema, desquamation, burning sensation, and steroid-induced telangiectasia. Less common AEs include acne/acne breakouts, hyperpigmentation, pruritus, skin atrophy, perioral dermatitis, and hypertrichosis [6-8].

This study aims to assess the attitude toward using Kligman’s formula and related outcomes among the general population in Saudi Arabia.

## Materials and methods

This is a cross-sectional study conducted among the general population of Saudi Arabia to assess the attitude toward using Kligman’s formula and related outcomes. A total of 292 participants were included in the study. A self-administered questionnaire was distributed among the targeted population using an online survey. The questionnaire includes socio-demographic characteristics, assessment of attitude, and satisfaction in using Kligman’s formula. Any individuals who are using Kligman's formula or have been using Kligman's formula among Saudi populations were included in this study. Incomplete questionnaires and participants who did not use Kligman's formula were excluded. Data were tabulated and cleaned in Microsoft Excel (Microsoft Corporation, Redmond, WA).

Statistical analysis

Data management and analysis were carried out using the Statistical Package for Social Sciences (SPSS) version 26 (IBM Corp, Armonk, NY). Descriptive statistics (mean, standard deviation, frequencies, and percentages) were used to quantify continuous and categorical study variables. A chi-square test was used to compare the level of attitude and satisfaction related to study variables. A p-value of <0.05 was considered statistically significant.

Ethical approval

All procedures that involved human participants were in accordance with the ethical standards of the institutional and/or national research committee and with the 1975 Helsinki Declaration and its later amendments or comparable ethical standards. The ethical approval was obtained from King Faisal University (reference number: KFU-REC-2021-OCT-EA00032).

## Results

A total of 292 respondents have been included in the study with a mean age of 26.9 years. The majority of the respondents were female (91.1%) and Saudi nationals (97.3%). The majority were single (68.5%) and had bachelor's degrees (71.9%). Students (46.2%) and unemployed (30.5%) constituted most participants. Only 12.3% worked in the healthcare sector, and 8.6% were doctors.

**Figure 1:**
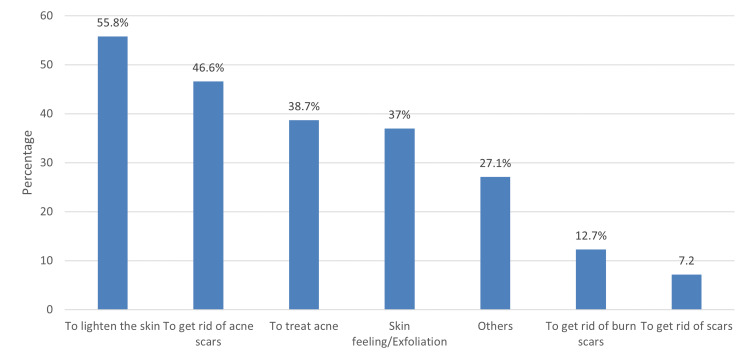
Reasons for using Kligman's formula.

The usage of Kligman’s formula among participants is referred to different aims (Figure 1), and skin lightening represents the most common one. However, the most common sources of information for using Kligman’s formula without a doctor's prescription were electronic social network sites (68.2%), followed by friends (22.9%) and websites (20.2%). At the same time, television/radio was the least (0.4%).

Of the participants, 38% estimated having a positive attitude in using Kligman’s formula, while 23.6% reported using Kligman’s formula according to doctor’s prescription. The prevalence of respondents who regularly visit dermatologists to guide them regarding Kligman’s formula was 17.1%. More than two-thirds (67.5%) were mixing the ingredients of Kligman’s formula by themselves. Of those self-preparing Kligman’s formula, 74.6% attempted to divide evenly without using any device. The proportion of respondents who bought a ready-made Kligman’s formula was 35.6%, while the proportion of respondents who experienced side effects due to Kligman’s formula was 50% (Table 1).

**Table 1:** Assessment of attitude toward the use of Kligman's formula (n = 292).

Study variables	N (%)
Current attitude of using Kligman's formula	
Positive	111 (38.0%)
Negative	181 (62.0%)
Currently using Kligman's formula based on doctor’s prescription	
Yes	69 (23.6%)
No	223 (76.4%)
Do you follow up regularly with your dermatologist to see how you use Kligman's formula?	
Yes	50 (17.1%)
No	242 (82.9%)
How often do you use Kligman's formula?	
Daily	114 (39.0%)
Every 2 days	71 (24.3%)
Every 3 days	54 (18.5%)
Every 2 weeks	23 (07.9%)
Every month	18 (06.2%)
Once in a while	12 (04.1%)
Timing of use for Kligman's formula	
In the morning	19 (06.5%)
In the evening	235 (80.5%)
In the morning and evening	38 (13.0%)
Duration of Kligman's formula use	
Less than a month	102 (34.9%)
1-2 months	81 (27.7%)
2-3 months	46 (15.8%)
3-6 months	22 (07.5%)
6-9 months	04 (01.4%)
9-12 months	05 (01.7%)
More than a year	32 (11.0%)
Did you collect and mix the ingredients for Kligman's formula yourself?	
Yes	197 (67.5%)
No	95 (32.5%)
How did you divide each product (what is the percentage of that product)? (n = 197)	
Tried to divide it evenly using the scale	22 (11.2%)
Tried to divide it evenly without using the scale and without any device	147 (74.6%)
Divide them differently	28 (14.2%)
Do you buy Kligman's formula ready-made?	
Yes	104 (35.6%)
No	188 (64.4%)
Experience any side effects after using Kligman's formula?	
Yes	146 (50.0%)
No	146 (50.0%)
Have you noticed any sensitivity in the skin of the face when exposed to the sun?	
Yes	13 (04.5%)
No	279 (95.5%)

**Figure 2:**
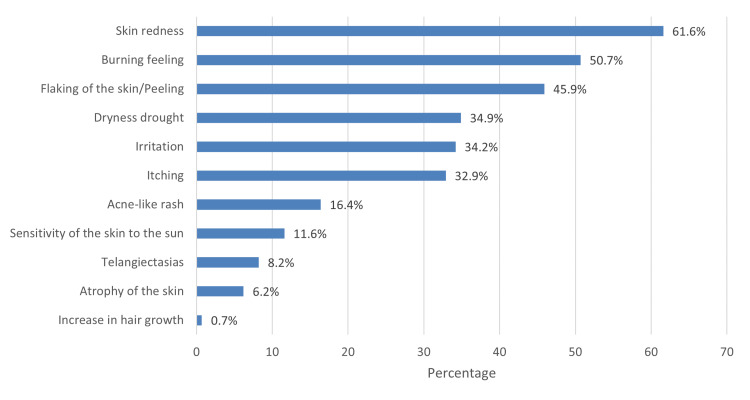
Side effects after using Kligman’s formula.

Respondents cited face as the most common body part where Kligman’s formula was applied (92.1%), followed by hand (14.7%) and knees (14.7%). It was further observed that the most frequently used ingredients were hydroquinone cream (Hiquin®) (64.5%), followed by tretinoin cream (Acretin®) (57.4%), and adapalene cream (Differin®) (49.2%). Moreover, the most commonly mentioned side effect of Kligman’s formula was skin redness (61.6%), followed by a burning sensation (50.7%) (Figure 2).

The overall mean satisfaction score was 6.02 (SD 2.89) out of 10 points with 53.1%, and 46.9% were classified into dissatisfied and satisfied, respectively. We also noted that 32.5% were still using Kligman’s formula even after obtaining a satisfactory result. After stopping from using Kligman’s formula, 44.9% expressed that they did not use anything, while 21.6% used other cream types instead. However, for the changes noticed after stopping the formula, 34.2% stated recurrence of the main problem, and 29.5% expressed persistence of the therapeutic result (Table 2).

**Table 2:** Satisfaction and outcome after using Kligman’s formula (n = 292).

Variables	N (%)
Satisfaction after using Kligman’s formula (mean ± SD)	6.02 ± 2.89
Dissatisfied (score ≤ 6)	155 (53.1%)
Satisfied (score > 6)	137 (46.9%)
Duration of Kligman’s formula use after reaching satisfaction	
Less than a month	88 (30.1%)
1-3 months	95 (32.5%)
3-6 months	28 (09.6%)
6-12 months	09 (03.1%)
I was not satisfied with the result	60 (20.5%)
Other	12 (04.1%)
If you stopped using Kligman's formula, what did you do after stopping?	
I used another type of cream	63 (21.6%)
I did not use anything	131 (44.9%)
I went to the dermatologist to treat the side effects	23 (07.9%)
I have not stopped yet	70 (24.0%)
Other	05 (01.7%)
What changes did you notice on your skin after you stopped using Kligman's formula?	
Maintaining the therapeutic result	86 (29.5%)
Repetition of the main problem	100 (34.2%)
Persistent side effects	10 (03.4%)
Worsening side effects	15 (05.1%)
I have not stopped yet	62 (21.2%)
No change in the treatment	19 (06.5%)

When measuring the relationship of the attitude and satisfaction among participants, it was found that positive attitude in using Kligman’s formula was more common among those using Kligman’s formula based on doctor's prescription (X^2^ = 20.030; p < 0.001), those who had regular follow up with dermatologist (X^2^ = 17.290; p < 0.001), and those who bought ready-made Kligman’s formula (X^2 ^= 5.679; p = 0.017), while negative attitude was more common among those who were using self-made Kligman’s formula (X^2^ = 4.119; p = 0.042).

## Discussion

The present study attempted to evaluate the attitude and satisfaction in using Kligman's formula while highlighting the adverse effect of misusage. The findings of this study revealed that only 38% exhibited a positive attitude and the rest (62%) had a negative attitude about it. Of those using Kligman's formula, 26% were using it according to the doctor's advice, with 17.1% of them having a follow-up visit with the dermatologist. In India [9], a study revealed a significant increase in the awareness of the long-term use of topical steroids, including Kligman's formula, with 55.2% of the medical students using it without prescriptions. The use of topical steroids without doctor’s prescriptions had been well-discussed in most publications. For instance, Jha et al. [10] reported that 42.9% of the patients bought topical corticosteroids (TC) containing creams over the counter without prescription, among them, 20% were recommended by their friends, family members, and neighbors, and 8.5% were recommended by a beautician. These findings corroborated the report of Sendrasoa et al. [11], which indicated that the majority of respondents (61%) obtained TC from cosmetic retailers, pharmacy stores (23%), and beauticians (12%), with only 0.26% using TC based on the physician’s prescription.

Alrayyes et al. [5] noted that skin lightening products could harm the skin since users were unaware of the product's active ingredients. Incidentally, in this study, due to the inappropriate use of Kligman's formula, half of the respondents had experienced adverse effects, including 4.5% reported photosensitivity of the face when exposed to sunlight. These results are in accordance with the study of Majid [8], indicating that complaints of side effects due to the Kligman's formula were seen among 26% of the patients. In a study conducted by Sendrasoa et al. [11], 13% of the Madagascar respondents planned to seek dermatological care due to cutaneous adverse effects after using TC. However, most of them were hesitant to proceed due to costly services.

Moreover, adverse reactions resulting from using the TC or combination therapy, including Kligman’s formula, may vary according to the type of cream or containing ingredients. It includes acne, hypopigmentation, pigmentation disorder, and cutaneous atrophy [10,11]. In our study, skin redness, burning feeling, and skin peeling were the most common side effects experienced by the respondents after using Kligman’s formula. While respondents in this study indicated adverse reactions when using the formula. However, nearly three-quarters (72.9%) were using sunscreen to reduce the side effect of the mixed ingredients or to protect against the dyspigmentation of the skin.

Siadat et al. [12] pointed out that combining modified Kligman’s cream + intense pulsed light yielded better satisfaction rates than modified Kligman’s alone. They further surmised that the combination showed better efficacy and faster response in the treatment of the post-burn hyperpigmentation without experiencing side effects after the treatment. Adverse reactions due to the application of the combined formula may vary according to the conditions to be treated. Therefore, it is essential to consult a doctor before using it.

The overuse of Kligman's formula has also been noticed in this study. In our observation, about one-third (32.1%) of the respondents were still using the combination therapy even after their satisfaction. Some indicated that it is necessary to maintain therapeutic results, while some of them used another type of cream after the discontinuation of Kligman’s formula. Moreover, more than one-third (34.2%) complained of conditions recurrence, which could be the main reason for the extended usage of the combined therapy. Similarly, Dhanalakshmi et al. [9] found that students used the topical medication beyond the prescribed period. A total of 52.6% had used the medication for a maximum of one month, and the most common reasons for continuation as cited by the medical students were the treatment had no effect (30%) or skin glowed after the application (23.3%).

The limitations of the present study include the sample size and self-reported attitude and side effects. In addition, the correlations are measured using a cross-sectional design, which may not provide a well-established casualty. Nevertheless, this study could be used as a baseline to further investigate this problem in the future.

## Conclusions

Kligman’s formula represents one of the therapeutic options for certain skin conditions, especially melasma and post-inflammatory hyperpigmentation. However, its application and preparation under medical advice with regular follow-up are crucial. Improper and uncontrolled prolonged formula usage could lead to different cutaneous side effects, including skin atrophy, acne, and rosacea.
